# Neurofeedback of Slow Cortical Potentials in Children with Attention-Deficit/Hyperactivity Disorder: A Multicenter Randomized Trial Controlling for Unspecific Effects

**DOI:** 10.3389/fnhum.2017.00135

**Published:** 2017-03-31

**Authors:** Ute Strehl, Pascal Aggensteiner, Daniel Wachtlin, Daniel Brandeis, Björn Albrecht, Maria Arana, Christiane Bach, Tobias Banaschewski, Thorsten Bogen, Andrea Flaig-Röhr, Christine M. Freitag, Yvonne Fuchsenberger, Stephanie Gest, Holger Gevensleben, Laura Herde, Sarah Hohmann, Tanja Legenbauer, Anna-Maria Marx, Sabina Millenet, Benjamin Pniewski, Aribert Rothenberger, Christian Ruckes, Sonja Wörz, Martin Holtmann

**Affiliations:** ^1^Institute for Medical Psychology and Behavioral Neurobiology, University of TübingenTübingen, Germany; ^2^Department of Child and Adolescent Psychiatry and Psychotherapy, Central Institute of Mental Health, Medical Faculty Mannheim, University of HeidelbergMannheim, Germany; ^3^Interdisciplinary Center for Clinical Trials at the University Medical Center of the Johannes Gutenberg University of MainzMainz, Germany; ^4^Department of Child and Adolescent Psychiatry and Psychotherapy, Psychiatric Hospital, University of ZurichZurich, Switzerland; ^5^Center for Integrative Human Physiology, University of ZurichZurich, Switzerland; ^6^Neuroscience Center Zurich, University of Zurich and ETH ZurichZurich, Switzerland; ^7^Child and Adolescent Psychiatry, University Medical Center GöttingenGöttingen, Germany; ^8^Department of Psychosomatic Medicine and Psychotherapy, University Hospital LeipzigLeipzig, Germany; ^9^Landschaftsverband Westfalen-Lippe (LWL) University Hospital Hamm for Child and Adolescent Psychiatry, Ruhr-University BochumHamm, Germany; ^10^Department of Child and Adolescent Psychiatry, Psychosomatics and Psychotherapy, University Hospital Frankfurt, Goethe UniversityFrankfurt am Main, Germany

**Keywords:** ADHD, neurofeedback, slow cortical potentials, randomized controlled study, EMG feedback, specificity

## Abstract

**Background:** Neurofeedback (NF) in children with attention-deficit/hyperactivity disorder (ADHD) has been investigated in a series of studies over the last years. Previous studies did not unanimously support NF as a treatment in ADHD. Most studies did not control for unspecific treatment effects and did not demonstrate that self-regulation took place. The present study examined the efficacy of NF in comparison to electromyographic (EMG) feedback to control for unspecific effects of the treatment, and assessed self-regulation of slow cortical potentials (SCPs).

**Methods:** A total of 150 children aged 7–9 years diagnosed with ADHD (82% male; 43% medicated) were randomized to 25 sessions of feedback of SCPs (NF) or feedback of coordination of the supraspinatus muscles (EMG). The primary endpoint was the change in parents’ ratings of ADHD core symptoms 4 weeks after the end of treatment compared to pre-tests.

**Results:** Children in both groups showed reduced ADHD-core symptoms (NF 0.3, 95% CI -0.42 to -0.18; EMG 0.13, 95% CI -0.26 to -0.01). NF showed a significant superiority over EMG (treatment difference 0.17, 95% CI 0.02–0.3, *p* = 0.02). This yielded an effect size (ES) of *d* = 0.57 without and 0.40 with baseline observation carried forward (BOCF). The sensitivity analysis confirmed the primary result. Successful self-regulation of brain activity was observed only in NF. As a secondary result teachers reported no superior improvement from NF compared to EMG, but within-group analysis revealed effects of NF on the global ADHD score, inattention, and impulsivity. In contrast, EMG feedback did not result in changes despite more pronounced self-regulation learning.

**Conclusions:** Based on the primary parent-rated outcome NF proved to be superior to a semi-active EMG feedback treatment. The study supports the feasibility and efficacy of NF in a large sample of children with ADHD, based on both specific and unspecific effects.

**Trial Register:** Current controlled trials ISRCTN76187185, registered 5 February 2009.

## Introduction

Attention-deficit/hyperactivity disorder (ADHD) is a common neurobehavioral disorder in childhood. According to DSM-IV-TR (in effect during this trial), core symptoms include impaired attention, hyperactivity, and impulsivity (American Psychiatric Association, 2000). Stimulant medication represents the most commonly used intervention for children with ADHD, but its use is limited since in some children pharmacotherapy may fail, adverse side effects are common, long-term effects are not yet established and some parents and clinicians have reservations about medication use (Sonuga-Barke et al., 2013).

Among additional or alternative treatments for ADHD neurofeedback (NF) has gained promising empirical support in recent years. Based on the observation of deviant slow event-related potentials in children with ADHD, feedback of slow cortical potentials (SCPs-NF) aims at improving the neurophysiological profile of children with ADHD by self-regulation of cortical excitation thresholds (Banaschewski and Brandeis, 2007; Albrecht et al., 2013; Doehnert et al., 2013). SCPs are slow event-related changes in the EEG, reflecting cognitive and motor preparation (Birbaumer et al., 1990). Studies have demonstrated promising effects on behavior, cognitive, and electrophysiological measures after SCP-NF (Heinrich et al., 2004; Strehl et al., 2006; Drechsler et al., 2007; Gevensleben et al., 2009; Christiansen et al., 2014; Maurizio et al., 2014).

A recent meta-analysis failed to support NF as an effective treatment for ADHD but this result may reflect methodological weaknesses of the available studies rather than the weakness of NF as such (Cortese et al., 2016). When the analysis was restricted to trials meeting Arns et al.’s (2014) criteria for a standard (established) NF protocol (as related to the target EEG measures, to number and placement of electrodes, trials designed in line with principles of learning theory, involving techniques to promote generalization to everyday life and assessing whether learning took place), significant effects emerged also applying probably blinded ratings.

The main drawbacks of previous SCP studies are methodological shortcomings like lack of appropriate control conditions, intent-to-treat analyses, unblinded outcome measures, limited testing for successful self-regulation at the brain level, and failure to control unspecific effects and variables (e.g., amount of reinforcement, time, attention of and interaction with the therapist; sex, age, baseline severity, expectations, and satisfaction with treatment). To disentangle NF-specific and unspecific effects influencing the outcome of any treatment the choice of a control condition is of major importance (Oken, 2008). *Active control* conditions do not control for unspecific effects as the independent variables causing them differ as regards, e.g., to setting, expectation, interaction, time, and effort. For example, medication cannot control for the unspecific effects of time and attention spent concentrating on the challenging self-regulation task, and for the experience of learning with contingent feedback. Double-blind studies which employ a sham condition may provide strong unbiased evidence regarding efficacy and specificity, and thus have clear merits in NF research (The Collaborative Neurofeedback Group, 2013) which may involve considerable non-specific effects (Drechsler et al., 2007; Thibault and Raz, 2016). While *double-blind controlled placebo* studies in general may provide strong evidence regarding efficacy and specificity, the establishment of sham conditions for NF treatments has shown to be at least doubtful if not missing the main aim. Patients and trainer can detect the sham condition and may refuse further participation (Birbaumer et al., 1991). Another outcome was observed by van Dongen-Boomsma et al. (2013). Here the majority of patients in the NF condition assumed that they were assigned to the sham condition. As any acquisition of a new skill, learning to self-regulate brain activity takes time. The lack of success in the first sessions may lead to the impression of being allocated to an ineffective control condition. As a consequence, this may impair motivation and compliance. However, apart from potential ethical and expectancy motivation problems of sham designs, an ideal control condition for NF should also require learning to fully match moderator variables such as motivation, frustration, compliance, and the often stepwise experience of self-efficacy (Gevensleben et al., 2014). Recent neuroimaging research demonstrates specific increases of activity in brain regions supporting inhibitory control following learning of different types of self-regulation in ADHD (Baumeister et al., 2016). Therefore, sham conditions that do not allow learning genuine contingencies also have limitations (Sherlin et al., 2011; Arns et al., 2014). To induce learning of self-regulation but limited to peripheral rather than central nervous targets, we chose a *semi-active control* condition according to the classification put forward by Arns et al. (2014) in comparing NF with electromyographic (EMG) feedback. Despite not being closely related to the known pathology of ADHD, EMG feedback has been used in several ADHD treatment studies and as a control condition for NF. Some improvements but smaller than those from NF were reported (Arnold, 2001; Bakhshayesh et al., 2011; Maurizio et al., 2014). Thus, even participants in the control condition have the chance to reduce symptoms and learn self-regulation, but not based on a treatment specific to the pathology of the disease. Delivering identical treatment elements in both conditions apart from the specific (NF or EMG) component should allow differentiating specific from unspecific effects of NF.

The aim of this investigation was to assess the effectiveness, specificity and feasibility of SCP-NF in comparison to EMG feedback in a prospective, randomized and controlled study, while neurophysiological data and more detailed learning analyses and correlations with clinical outcomes will be published elsewhere (see Materials and Methods).

## Materials and Methods

### Study Design

**FIGURE 1:**
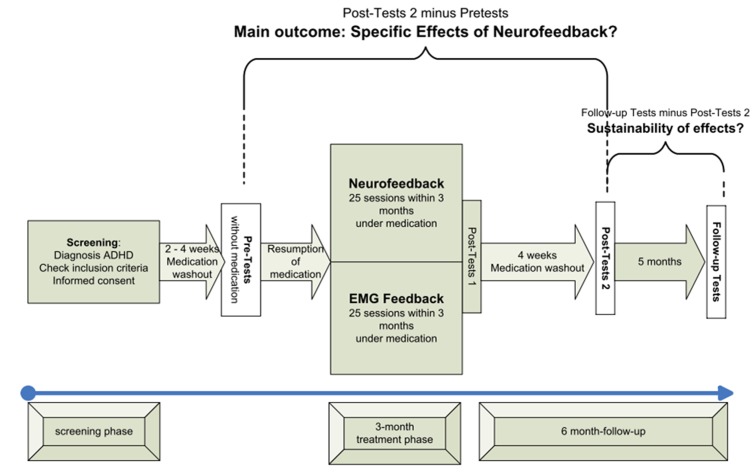
**Study flow (modified from Holtmann et al., 2014)**.

Study design, methods, and data analysis plan are described in detail in the study protocol published by Holtmann et al. (2014). Patients were recruited and treated in five German university-based outpatient departments for child psychiatry/psychotherapy. All local ethics committees approved the study. Patients’ assent was obtained by using age appropriate information and their parents or guardians gave written informed consent. **Figure 1** depicts the design and study flow.

### Study Groups

Inclusion criteria comprised age from 7 to 9 years, and a diagnosis of ADHD combined type according to DSM-IV TR verified in a semi-structured interview under the supervision of clinical psychiatrists/licensed psychologists (Delmo et al., 1998). In the case of positive screenings for comorbid symptoms assessed by the Child Behavior Checklist (CBCL; Arbeitsgruppe Deutsche Child Behavior Checklist, 1998), corresponding parents’ rating scales were applied (Döpfner and Lehmkuhl, 2000; Döpfner et al., 2006). Exclusion criteria consisted of a diagnosis of bipolar disorder, psychosis, serious obsessive–compulsive disorder, chronic severe tics or Tourette syndrome, major neurological or physical illness, acute suicidal tendencies, pharmacotherapy for severe anxiety, mood disorders and psychosis, IQ below 80, lack of German-language proficiency in the child or primary caregiver, no telephone, pregnancy and lactation, and current participation in other clinical trials.

As the interventions were considered an add-on to treatment as usual pharmacotherapy for ADHD, Oppositional Defiant Disorder and Conduct Disorder were allowed.

Patients were randomly assigned in a 1:1 ratio with varying block size to either the experimental or the control group. This assignment was realized by a computer-generated, web-based tool provided by the Interdisciplinary Center for Clinical Trials (IZKS) Mainz. Randomization was stratified per trial site and sex. Medical consultants rating clinical impairments were blinded. Participants were not blinded, because they were instructed according to their group assignment. Parents were not informed about treatment allocation but as the children were given instructions according to their treatment group, parents could infer their child’s group assignment.

### Procedures

After screening, there was a washout period of 2 weeks for children with psychostimulants and 4 weeks for participants with atomoxetine. Pre-tests and post-tests 2 were conducted without medication. After the pre-test, children resumed their medication until completion of post-tests 1. The main outcome therefore was assessed by changes in post-tests 2 compared to pre-tests (see **Figure 1**).

Participants received 25 training sessions within 3 months with two to three sessions per week. After session 12, there was a break of 4–6 weeks. Such a break has become standard in clinical NF studies to disburden the patients from the demanding training schedule with two to three sessions per week and to give him/her the opportunity to practice self-regulation in everyday life (transfer).

#### Experimental Group: NF of SCP

SCPs are very slow shifts in the EEG near to 0 Hz, typically generated in an event-related design for several seconds. A negative shift reduces the excitability of the underlying cortical area while a positive shift is understood as inhibition of excitation and/or consummation of energy. As event-related potentials they prepare adequate cognitive as well as motor responses. In the feedback paradigm, participants were prompted to either produce negative (reducing the excitability threshold of the underlying cortex) or positive shifts (inhibition of excitation) in a randomized order. After session 12, the ratio of negativity to positivity trials was increased from 50 to 80%. The convention in SCP training so far has been to train and reinforce both polarities to improve self-regulation, but particularly toward the end focus on that polarity which is thought to be related to the disease (e.g., Strehl et al., 2006). As the neurophysiological profile of patients with ADHD indicates hypoactivation of cortical excitation thresholds, the training of negative shifts is thought to be more important.

#### Control Group: Feedback of Electromyographic Coordination of the Supraspinatus Muscles

As a semi-active control condition EMG feedback of coordination in the supraspinatus muscles was chosen. Participants were instructed either to contract or to relax the left relative to the right supraspinatus muscle. This protocol was chosen to induce differential EMG control corresponding to the “polarities” comparable to the NF condition, without requiring simple relaxation or tension. This allowed us to use the same device and the same representation of the feedback signal on the screen. We did not choose a standard EMG feedback protocol because the control condition should be as unspecific as possible but include the possibility to learn self-regulation, i.e., the unspecific variable of any biofeedback treatment.

#### Common Components of Behavior Therapy in Both Groups

All interventions took place in outpatient clinics. Setting, training devices, electrode montage, feedback and transfer trials, number of sessions, transfer exercises, and the possibility to earn tokens were the same in both groups.

**FIGURE 2:**
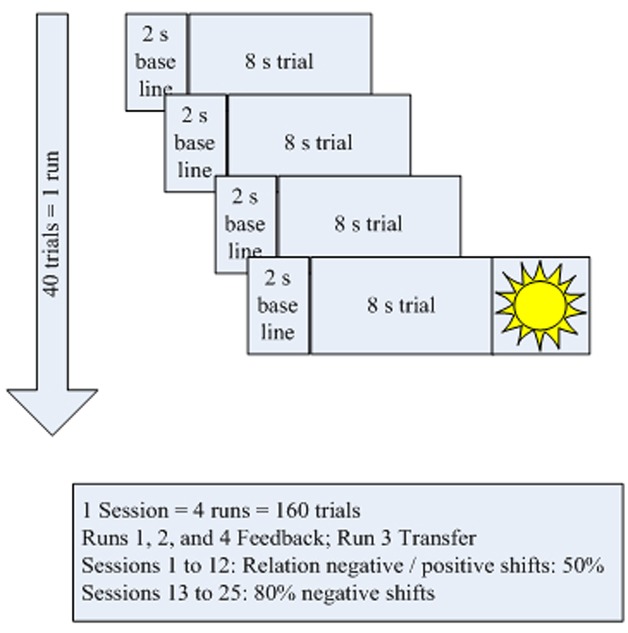
**Treatment schedule**.

The treatment schedule (**Figure 2**) for each session comprised four blocks of 40 trials each. Each trial lasted for 10 s (2 s baseline and 8 s feedback and depicting a “sun” after successful trials). In all sessions, the third block operated without continuous feedback; only the sun was shown at the end if the trial had been successful (**Figure 2**). These trials were part of several measures to carry over self-regulation skills to everyday life:

During the break following session 12, patients were asked to practice self-regulation at home using small memo cards depicting the screen during a task. In addition, self-regulation could be trained with the help of a video showing a sequence of both positivity and negativity trials. After each of the 10 final sessions, the children did part of their homework in the lab under the trainer’s supervision making use of the memo cards.

As a reward for their participation and for good cooperation children could earn tokens. Whenever a certain number was achieved, tokens were swapped for vouchers or small gifts.

### Acquisition of EEG- and EMG-Signals

EEG and EMG were recorded and fed back with a multichannel amplifier (THERA PRAX^®^ neuroConn GmbH, Ilmenau, Germany). The EEG electrode was placed at Cz, referenced against the mastoid behind the right ear. Four electrodes were used to record the vertical and horizontal electrooculogram (EOG) and one electrode behind the left ear was used as ground. EMG was recorded with one electrode per shoulder placed at the upper area of the right and left supraspinatus muscle.

Off-line analyses were performed with the Brain Vision Analyzer (Version 2.0, Brain Products, Gilching, Germany). For training data, EEG was filtered off-line using a 0.01 Hz high cut-off filter (12 dB/octave) plus 50 Hz notch filter, referenced with one mastoid, followed by ocular correction (Gratton et al., 1983). Data were segmented for both tasks (positive and negative SCP shifts). Artifacts were rejected semi-automatically if trials were over ±150 μV. Remaining artifact free trials were averaged. The average was exported using the last 4–8 s of every trial that lasted 8 s.

Each center was guided by a detailed manual to ensure equal handling of participants, testing, and treatment. Center representatives met for an initial 2-day training course and on a regular basis thereafter. Supervision visits took place to guarantee compliance with the manual.

### Outcomes

Psychometric properties of all pre-specified measures are reported in Holtmann et al. (2014). For the present first paper outcomes are reported as changes from pre-test to post-test 2 (after washout of medication, defined *a priori* as the primary endpoint to avoid residual medication confounds, and to focus on stable or sustained SCP-NF effects). Apart from IQ and cortical self-regulation, a detailed analysis of learning parameters, electrophysiological, neuropsychological outcomes, and 6-month follow-up data will be reported elsewhere.

The primary endpoint was the parent-rated ADHD symptom severity assessed using the mean global score of the German ADHD rating scale (24). The scale consists of 20 items assessing the severity of inattention, hyperactivity, and impulsiveness. Each item, corresponding to one of the DSM-IV diagnostic criteria, is rated on a 4-point scale (0 = never or rarely; 1 = sometimes; 2 = often; 3 = very often).

Secondary endpoints were:

– Parents’ ratings of ADHD subdomains (inattention, hyperactivity, impulsivity; Döpfner et al., 2006).
– Teachers’ ratings of ADHD symptoms (global score and subdomains; Döpfner et al., 2006).
– The Clinical Global Impression-Improvement (CGI-I) responder status assessed by a blinded clinician (Guy, 1976).
– Comorbid symptoms [parents’ and teachers’ ratings via the Strengths and Difficulties Questionnaire (SDQ); Woerner et al., 2004].
– Full scale IQ [indicated by its percent rank; measured with parallel versions of the Coloured Progressive Matrices (CPM) to minimize test–retest effects; Bulheller and Häcker, 1998.
– Quality of life assessed via the global score of the revised German Kid-KINDL(R) (Ravens-Sieberer and Bullinger, 1998).
– Parents’ satisfaction with therapy: unpublished questionnaire developed by the Institute of Medical Psychology and Behavioral Neurobiology, Tübingen (2004). Parents submitted these questionnaires directly to the IZKS to guarantee anonymous handling and thereby avoiding responses driven by social desirability.
– Adverse events (AE) and serious adverse events (SAE): at each contact participants were asked to report any AE and their severity using open questions.

As covariates, we assessed parenting style and parents’ expectations (Arnold et al., 1993) at screening. Self-regulation of EEG during training sessions was assessed to evaluate the specificity of NF.

### Statistical Analysis

The methodology for processing and analyzing the data was documented in a Statistical Analysis Plan (SAP) dated and maintained by the IZKS responsible for data management, monitoring, and analysis (for details, see Holtmann et al., 2014). Sample size calculation for the primary endpoint was based on an estimation of effect sizes derived from the SCP-NF study by Heinrich et al. (2004) using the same outcome measure. Expecting a mean ADHD score of 1.2 at post-test 2 in the NF group and of 1.5 in controls with a common SD of 0.55 a sample size of 72 per group was required to achieve a power of 90% (α = 0.05, two-sided *t*-test).

Data were analyzed primarily in the modified intention-to-treat (mITT) population, comprising all randomized patients except those who received no treatment due to violation of inclusion criteria. Supportive analysis was performed in the per-protocol (PP) population, comprising all mITT patients who did not meet any of the following criteria: violation of inclusion or exclusion criteria, major deviations from the visit schedule, and lack of compliance during treatment sessions. Safety parameters were analyzed in the safety population, comprising all participants with at least one feedback session.

The primary outcome was tested by an analysis of covariance (ANCOVA) with treatment, trial site, sex, baseline ADHD score, baseline ADHD medication, parenting style, and parents’ expectations as covariates. Missing ADHD scores were imputed according to the baseline observation carried forward (BOCF) method. This is usually considered a conservative approach to handle missing data since patients with missing values are treated as treatment failures. This conservative approach is supposed to avoid too positive results, when many patients from the NF treatment group dropout who do not improve or even get worse. Therefore, the analysis was repeated with a multiple imputation approach. Additional covariates were used to create 10 complete datasets. Those datasets were analyzed by the same ANCOVA model as the primary analysis. Afterward, the results were combined by Rubin’s rules. Secondary analyses comprised ANCOVAs (analogously to the primary analysis) for differences in ADHD global and subdomain scores (teachers’ ratings), *t*-tests for differences in ADHD global scores (parents’ ratings, teachers’ ratings), SDQ subscales, IQ, quality of life, and parents’ satisfaction with therapy. For the binary variable Clinical Global Impression (CGI) McNemar’s tests were used to test for differences between time points within groups, and chi-squared tests were used for differences between groups. Results of all statistical tests except for the primary analysis must be interpreted in an exploratory manner. To assess the magnitude of treatment effects, between-treatment effect sizes were calculated by dividing the treatment-group differences (including the BOCF method if indicated) by the pooled standard deviation at pre-test. Within-treatment effect sizes were calculated by dividing the mean of changes by the standard deviation at pre-test.

To assess the extent and specificity of SCP self-regulation, the mean amplitude of SCPs and mean self-regulation performance (percentage of correct trials) were averaged for training sessions 2 + 3, 10 + 11, 14 + 15, and 23 + 24. These session averages were selected in line with previous work (Strehl et al., 2006). This selection provides robust estimates of regulation performance and learning, while excluding the undesired influences of novelty or expected completion in the initial and final sessions of each training half. SCP amplitude (μV) was analyzed using group by task × sessions (only the four session averages during training to minimize the number of dropouts) repeated-measures ANOVAs. Self-regulation performance was analyzed using a group by sessions (the four session averages during training plus post 2 performance) repeated-measures ANOVA. SCP amplitude and self-regulation performance were analyzed separately for the feedback and transfer condition.

An independent data monitoring and safety board supervised the conduct of the study. The trial was registered under Current Controlled Trials ISRCTN76187185 (5 February 2009).

## Results

### Patients

**FIGURE 3:**
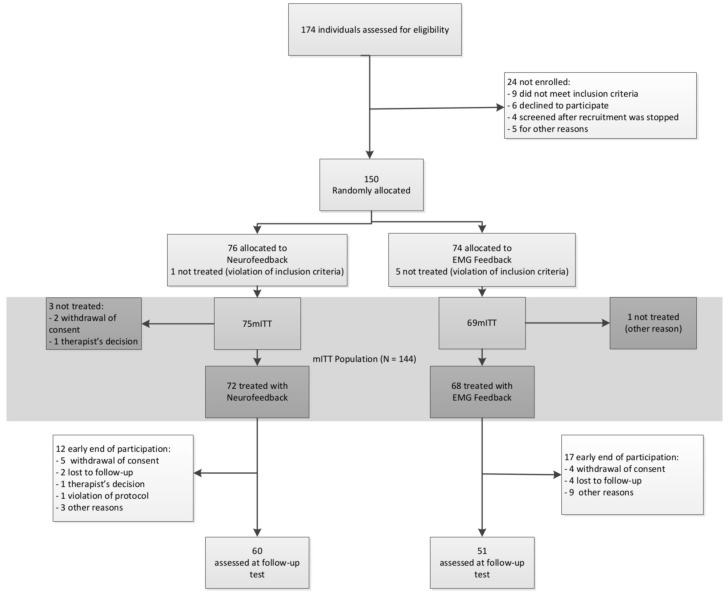
**CONSORT flow diagram**.

A total of 174 participants were recruited between September 2009 and January 2013 for screening, 150 of whom were allocated to one of the two treatment groups. In NF 60 and in EMG 51 participants completed treatment and took part in all assessment points. The CONSORT flow diagram is depicted in **Figure 3**.

The mITT population comprised 75 participants in NF and 69 in EMG. Baseline characteristics are depicted in **Table 1**. There were no differences between groups in any of these variables.

**Table 1:** Baseline characteristics of participants.

	Neurofeedback *N* = 75		EMG feedback *N* = 69	
Variable	Mean	*SD*	Mean	*SD*
Age (in years)	8.60	0.92	8.57	0.88
ADHD global score	1.84	0.45	1.78	0.47
KINDL(R) Quality of Life	67.5	8.9	68.6	9.6
CPM (percentage rank)	63.4	27.0	65.5	27.0
CBCL *t*-value				
Global	63.6	8.4	63.2	7.8
Externalizing problems	66.3	9.4	64.8	9.4
Internalizing problems	62.2	9.5	62.4	9.3

	***N***	**Percent**	***N***	**Percent**

CBCL Comorbidity^∗^				
Oppositional defiant disorder	31	41.33	32	46.36
Conduct disorder	0	0.00	1	1.45
Depression	11	14.66	8	11.59
Dysthymia	5	6.67	3	4.35
Separation anxiety	3	4.00	5	7.25
General anxiety disorder	18	24.00	18	27.69
Social phobia	4	5.33	8	11.59
Specific phobia	4	5.33	6	8.64
Sex				
Female	14	18.67	11	15.94
Male	61	81.33	61	84.06
CGI-S				
Normal/Borderline ill	3	5.00	3	5.36
Mild/Moderately ill	29	48.33	29	45.78
Marked/Severely ill	28	46.67	24	42.86
Missing	15		13	
ADHD medication prior to study	34	45.33	28	40.58

*CGI, Clinical Global Impression-Severity; CBCL, Child Behavior Checklist; ^∗^disorder-specific parent ratings.*

The safety population comprised 96% of the mITT population for NF and 98.55% for EMG; the PP population consisted of 59% for NF and 58% for EMG. The main reason for violation of the protocol was delay of post-tests 2, which occurred in 41% of NF and in 42% of EMG mITT populations. NF had 16% dropouts, EMG 17%, with most dropouts occurring between pre-test and session 12. A comparison between dropouts and non-dropouts yielded the following differences: lower level of education of fathers (*p* = 0.03—chi-squared test), fewer parents living together (*p* = 0.027—chi-squared test), and more severe oppositional defiant disorder (*p* = 0.033—*t*-test) in those who did not complete the study.

### Primary Outcome

NF was significantly superior to EMG in reducing ADHD core symptoms as rated by parents with a difference of 0.17 (95% CI 0.02–0.30, *F*(1) = 5.30, *p* = 0.02). ANCOVA yielded no impact of sex, trial site, medication status at baseline, parenting style, and parents’ expectations on the reduction of ADHD core symptoms as rated by parents (**Table 2**). The more pronounced ADHD symptoms were at pre-test the larger was their reduction.

**Table 2:** Primary analysis: Differences in ADHD global score (parents’ ratings; post-test 2 minus pre-test between groups; mITT population, ANCOVA, baseline observation carried forward); df, degree(s) of freedom.

	Adjusted mean (95% CI)	*p*	*F*	*df*
EMG feedback	-0.1338 (-0.259/-0.008)			
Neurofeedback	-0.2987 (-0.416/-0.181)			
Difference between treatments	0.1649 (0.023/0.301)			
Treatment		0.0230	5.30	1
Baseline ADHD global score		0.0008	11.84	1
Sex		0.1879	1.75	1
Trial site		0.5951	0.70	4
Baseline ADHD medication (yes/no)		0.3016	1.08	1
Parenting style (mean)		0.8007	0.06	1
Parents’ expectations (mean)		0.4154	0.67	1

**FIGURE 4:**
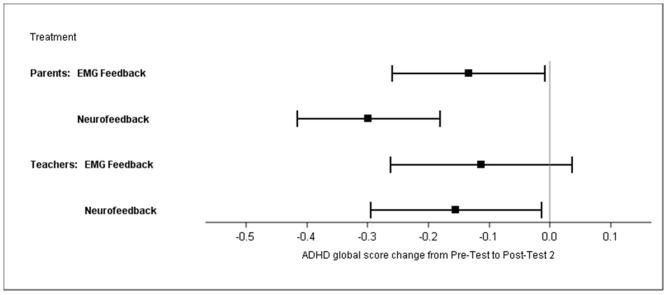
**Least square means of ADHD global score changes from pre-test to post-test 2 (parents’ and teachers’ ratings; mITT population, ANCOVA, baseline observation carried forward)**.

The sensitivity analysis with the PP population (*N* = 84) yielded similar results (Supplementary Table S1). The multiple imputation approach revealed similar results: the difference between treatments was 0.22 (95% CI 0.03–0.4), *p* = 0.02. The difference of changes in the ADHD global score between groups, as compared by a *t*-test, was significant for the mITT population (BOCF), at *p* = 0.01 (NF mean -0.35, SD 0.42; EMG mean -0.17, SD 0.43), and yielded an ES of *d* = 0.57 without BOCF and 0.40 with BOCF. Within-group analyses revealed effect sizes of 0.78 for NF and 0.35 for EMG. Global score changes from pre-test to post-test 2 are depicted in **Figure 4**.

### Secondary Outcomes

#### Parents’ Ratings of ADHD Subdomains

Data for all scales at pre-test and post-test 2 are reported in the **Table 3** (BOCF). For results without BOCF, see Supplementary Table S2.

**Table 3:** Parents’ ADHD ratings (mITT population *N* = 144, BOCF).

	NF		EMG		Total	
	Pre-test	Post-test 2	Pre-test	Post-test 2	Pre-test	Post-test 2
**Hyperactivity**						
*N*	72	73	67	68	139	141
Mean (*SD*)	1.54 (0.63)	1.22 (0.71)	1.52 (0.67)	1.33 (0.66)	1.53 (0.64)	1.28 (0.68)
Missing	3	2	2	1	5	3
**Impulsivity**						
*N*	72	73	67	68	139	141
Mean (*SD*)	1.93 (0.65)	1.59 (0.65)	1.80 (0.78)	1.71 (0.76)	1.87 (0.73)	1.65 (0.71)
Missing	3	2	2	1	5	3
**Inattention**						
*N*	72	73	67	68	139	141
Mean (*SD*)	2.03 (0.53)	1.64 (0.59)	1.97 (0.51)	1.80 (0.48)	2.00 (0.52)	1.72 (0.54)
Missing	3	2	2	1	5	3
**Global score^∗^**						
*N*	72	73	67	68	139	141
Mean (*SD*)	1.84 (0.49)	1.49 (0.55)	1.78 (0.47)	1.62 (0.50)	1.81 (0.46)	1.55 (0.53)
Missing	3	2	2	1	5	3

*^∗^Global score could not be assessed if more than two items in subscales were missing.*

**Table 4** shows the adjusted mean difference of change scores post-test 2 minus pre-test between groups (BOCF). Both groups improved in the subdomain hyperactivity, although no difference between groups was observed. Parents’ ratings indicated superior improvements in the NF group for the subscales impulsivity (*p* = 0.02) and inattention (*p* = 0.02) with medium effect sizes. Similar to the primary analysis none of the covariates had an impact on treatment differences.

**Table 4:** Adjusted mean differences in ADHD subdomain scores (parents’ ratings; post-test 2 minus pre-test between groups; mITT population, ANCOVA, BOCF).

	Neurofeedback *N* = 75 (23 BOCF)		EMG feedback *N* = 69 (20 BOCF)		Difference			
			
Variables	Mean	95% CI	Mean	95% CI	Mean	95% CI	*p*	ES
Hyperactivity	−0.28	−0.42/-0.13	0.17	−0.33/-0.02	0.11	−0.07/0.28	0.23	0.18
Impulsivity	−0.30	−0.45/-0.15	−0.09	−0.25/0.07	0.21	0.03/0.39	0.02	0.34
Inattention	−0.31	−0.44/-0.18	−0.13	−0.27/0.01	0.18	0.03/0.36	0.02	0.40

#### Teachers’ Ratings of ADHD Core Symptoms

The difference between groups based on teachers’ ratings of ADHD global scores (mITT population, ANCOVA, BOCF; Supplementary Table S3) was not significant [treatment difference 0.04, 95% CI -0.12 to 0.21, *F*(1) = 0.25, *p* = 0.62]. ANCOVA yielded a significant within-group difference for NF (mean change of -0.16; 95% CI -0.3 to -0.02) but not for EMG (mean change of -0.11; 95% CI -0.26 to 0.04). Data for all scales at pre-test and post-test 2 are reported in **Table 5** (BOCF); for results without BOCF, see Supplementary Table S4.

**Table 5:** Teachers’ ADHD ratings (mITT population *N* = 144, BOCF).

	NF		EMG		Total	
	Pre-test	Post-test 2	Pre-test	Post-test 2	Pre-test	Post-test 2
**Hyperactivity**						
*N*	68	70	63	64	131	134
Mean (*SD*)	1.15 (0.81)	1.05 (0.79)	1.02 (0.85)	1.02 (0.77)	1.09 (0.83)	1.04 (0.78)
Missing	7	5	6	5	13	10
**Impulsivity**						
*N*	68	70	63	64	131	134
Mean (*SD*)	1.41 (0.95)	1.24 (0.94)	1.31 (0.95)	1.27 (0.92)	1.36 (0.95)	1.25 (0.93)
Missing	7	5	6	5	13	10
**Inattention**						
*N*	68	70	63	64	131	134
Mean (*SD*)	1.69 (0.70)	1.59 (0.70)	1.68 (0.72)	1.60 (0.68)	1.69 (0.71)	1.60 (0.69)
Missing	7	5	6	5	13	10
**Global score^∗^**						
*N*	65	69	60	61	125	130
Mean (*SD*)	1.48 (0.64)	1.34 (0.68)	1.38 (0.71)	1.32 (0.71)	1.43 (0.67)	1.33 (0.66
Missing	10	6	9	8	19	14

*^∗^Global score could not be assessed if more than two items in subscales were missing.*

*Post hoc* *t*-tests for changes from pre-test to post-test 2 in global score and subscores yielded no differences between groups (see **Table 6**). According to within-group analyses, improvements in global scale, inattention, and impulsivity were observed for NF only, albeit with small effect sizes.

**Table 6:** Mean differences (SD) in ADHD global and subdomain scores (teachers’ ratings; post-test 2 minus pre-test between and within groups; mITT population, BOCF).

	Within group analysis									
	Neurofeedback *N* = 75 (26 BOCF)				EMG feedback *N* = 69 (28 BOCF)				Between groups analysis	
Variables	Mean	*SD*	*p*	ES	Mean	*SD*	*p*	ES	*P*	ES
Hyperactivity	−0.11	0.70	0.22	0.13	−0.01	0.56	0.86	0.01	0.40	0.11
Impulsivity	−0.20	0.70	0.03	0.21	−0.06	0.62	0.45	0.06	0.24	0.15
Inattention	−0.13	0.53	0.04	0.19	−0.08	0.43	0.16	0.11	0.51	0.08
Global score^∗^	−0.15	0.54	0.03	0.23	−0.07	0.41	0.19	0.10	0.36	0.12

*^∗^NF: N = 47; EMG: N = 39 because global score could not be assessed if more than two items in subscales were missing.*

#### Clinical Global Impression

Clinicians did not observe significant differences between groups regarding the responder status. At post-test 2 the percentage of responders was 27% (NF) and 26% (EMG). The analysis was limited due to a large proportion of missing values (about 40% of the mITT population in both groups).

#### Comorbid Symptoms (SDQ)

No difference between groups was observed regarding changes in comorbid symptoms between pre-test and post-test 1, as assessed with parents’ ratings. Children were rated as slightly improved in both groups.

#### Full Scale IQ (CPM)

A significant difference between groups was observed regarding the change in full scale IQ from pre-test to post-test 2 (*p* = 0.04, ES = -0.37). While the percentage rank in the EMG group declined from pre- (mean 65.5, SD 25.7) to post-assessment (mean 59.9, SD 31.4) it improved in the NF group from pre- (mean 63.4, SD 28.0) to post-assessment (mean 65.7, SD 28.0).

#### Quality of Life [KINDL(R)]

There was no change from pre-test to post-test 2. Scores in both groups ranged from 68 to 72, which is below the standard values of healthy children (Ravens-Sieberer and Bullinger, 1998).

#### Parents’ Satisfaction with Treatments

There were no differences in parents’ ratings regarding their satisfaction with the treatment. Mean values were 4.1 (SD 1.6) for NF and 4.4 (SD 1.4) for EMG on the 6-point Likert scale.

#### Adverse Events and Serious Adverse Events

In the safety population (*N* = 140) 119 AE were reported. At least one AE was reported in 33% of NF participants and 35% of EMG participants. A possible causal relation with the treatment was stated in 4 (6%) of NF participants and 5 (7%) of EMG participants. These children reported headaches (*N* = 4, both groups), skin reactions (*n* = 3, NF), myalgia (*n* = 1, EMG), and nausea (*n* = 1, EMG). SAE were reported for two children in each group (deterioration of ADHD: *n* = 2, EMG; *n* = 1, NF; psychological trauma after traffic accident: *n* = 1, NF). One of these children (EMG group) was withdrawn from the study because ADHD symptoms deteriorated.

#### Self-Regulation of EEG

**FIGURE 5:**
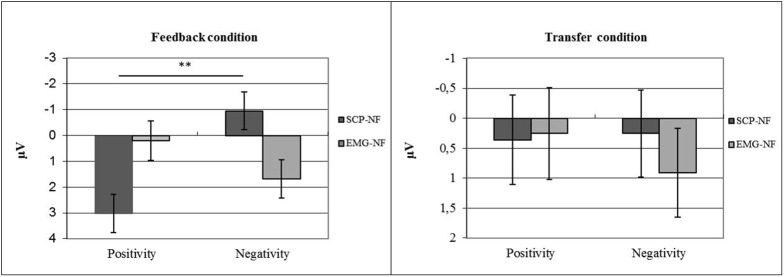
**Self-regulation of SCP amplitude by group (NF vs. EMG) and task (polarity; positivity vs. negativity)**.

For the SCP amplitude averaged over all training sessions, a significant interaction was observed between shift direction (trial polarity) and group (*p* ≤ 0.0001, η^2^ = 0.18). Only the SCP-NF group differentiated between EEG polarities (*p* < 0.0001), achieving negative mean amplitudes in negativity trials and positive amplitudes in positivity trials. These correct polarities were only achieved in the feedback condition, while the transfer condition did not show significant differences between polarities or groups (see **Figure 5**).

**FIGURE 6:**
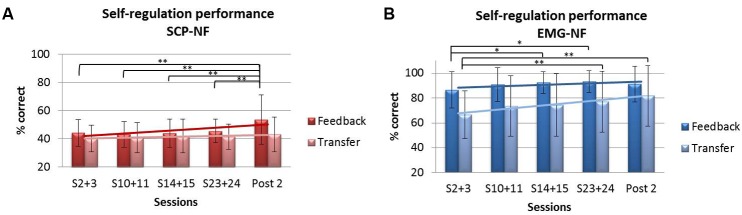
**Self-regulation performance during feedback and transfer trials: (A)** SCP-NF group and **(B)** EMG-NF group.

Repeated-measures ANOVA for self-regulation performance during feedback trials revealed a significant main effect of session (*p* < 0.001, η^2^ = 0.067) and a group × session interaction (*p* < 0.006, η^2^ = 0.054). The EMG-NF group achieved higher self-regulation rates compared to the SCP-NF group (*p* < 0.0001). *Post hoc* comparisons showed that the SCP-NF group improved significantly self-regulation at post 2, and the EMG group improved performance over sessions, however, there the last session was not different from the first one. For the transfer condition, repeated-measures ANOVA showed a significant main effect of session (*p* < 0.001, η^2^ = 0.044) but no group × session interaction. The EMG group achieved higher self-regulation rates compared to the SCP-NF group (*p* < 0.0001). *Post hoc* comparisons showed that only the EMG group enhanced performance over time (see **Figure 6**).

#### Self-Regulation Performance and its Relation to Clinical Changes

To assess the impact of self-regulation performance on the clinical outcome we grouped participants into learners and non-learners based on the sign of their regression slope for the feedback and the transfer condition separately. For the feedback condition 67.9% were classified as learners in the SCP-NF group, while 71.1% in the EMG group were classified as learners. For the transfer condition, 53.7% of the SCP-NF group and 73.7% of the EMG group were classified as learners.

No significant correlation between performance and clinical outcome was obtained for either group on the primary parent-rated outcome or the corresponding secondary teacher rated total score.

#### *Post hoc* Analyses

Response status was defined based on CGI; however, there were too many missing data for the analysis. We therefore assessed the responder rates based on a >25% improvement on the parent rated ADHD global score from pre-test to post-test 2. As a result, NF yielded a responder rate of 52% and EMG of 35% (mITT population). Based on BOCF analysis we observed 38% responder after NF and 25% after EMG feedback.

To explore possible reasons for the difference between parents’ and teachers’ ratings we computed an independent samples *t*-test (Satterthwaite). Teachers rated symptoms as less severe than parents did (see **Table 7**).

**Table 7:** Comparison of parents’ and teachers’ ratings ADHD global scale between groups.

	Parents		Teachers		*P*
	Mean	*SD*	Mean	*SD*	
NF pre-test	1.84	0.45	1.48	0.64	0.0002
NF post-test 2 minus pre-test	−0.49	0.42	−0.21	0.54	0.01
EMG pre-test	1.78	0.47	1.38	0.71	0.0003
EMG post-test 2 minus pre-test	−0.27	0.50	−0.11	0.51	0.28

## Discussion

This is the first randomized controlled clinical trial to investigate the specificity of SCP feedback in children with ADHD, and the largest study on an outpatient ADHD sample treated with NF. We compared two treatments (SCP-NF and EMG feedback) using identical training setups to control for unspecific effects. For the first time in NF research a BOCF approach was used to handle missing data. This study confirmed specific positive effects of SCP-NF on parent-rated ADHD symptom severity, with a significant greater decrease in symptoms compared to EMG feedback. Sex, trial site, medication, parenting style, and parents’ expectation had no impact on the ADHD score change. Sensitivity analyses with multiple imputation and with the PP population generated comparable results. These results are in line with previous findings of trials comparing NF to semi-active control groups (Arns et al., 2014). Symptom severity, comorbidity pattern, and age of our sample match that of the gold-standard MTA-study and can be regarded representative for children referred for outpatient ADHD treatment (The MTA Cooperative Group, 1999).

Our study set out to assess both specific and unspecific effects of NF. An important hint for specificity is the demonstration of successful SCP self-regulation for children in the SCP-NF group only. The significant symptom improvement in NF may be regarded as a confirmation of specific effects of SCP-NF. The lack of SCP regulation during the transfer condition in the NF group may suggest either limited or delayed transfer and a restricted generalization into everyday life. Here we should wait for follow-up results as it was shown previously for patients with epilepsy (Kotchoubey et al., 1997) and children with ADHD (Strehl et al., 2006) that performance in transfer trials improved substantially 6-month after the end of training.

In addition, our results also point to a strong influence of unspecific variables on treatment outcome.

We compared two treatments using identical conditions regarding tasks, time schedule, possible amount of reinforcement, and interaction. Children in the semi-active control group underwent the same intense treatment in an identical setting. In feedback treatments, contingent reinforcement of regulation of a physiological parameter improves self-efficacy and coping (Holroyd et al., 1984). Thus, EMG feedback may have an impact on ADHD symptoms (by improving self-regulation skills) even though there is no known direct relation between control of EMG activity and the neurobiological pathology of ADHD. It has to be noted that the type of EMG feedback used in this study is different from the EMG feedback protocols used previously in a couple of studies in the treatment of ADHD (for a review, see Arnold, 2001), showing some effects compared to conditions such as sham feedback, waiting list, keeping children busy by just playing or listening to a story-teller. Bakhshayesh et al. (2011) who used EMG feedback as a control condition for theta/beta-feedback report some but smaller effects for EMG feedback compared to frequency band NF. A comparison of tomographic NF with EMG feedback yielded only small differences between treatments with a tendency for EEG feedback with better improvements (Maurizio et al., 2014). While the former study trained relaxation of muscles our participants had to succeed in the simultaneous relaxation and tension of two different muscles similar to the latter study. This differential EMG feedback is far away from standard EMG feedback relaxation protocols aiming to reduce hyperactivity, a core symptom of ADHD. It therefore should be of limited specific influence on ADHD symptoms. The finding of similarly reduced hyperactivity according to parents’ ratings in both groups fits into this consideration of unspecific effects.

The rather small (0.40 with BOCF) or medium (0.57 completer) effect size of the between group comparisons should be discussed in several respects. First, due to the considerable unspecific effects in the semi-active control group the clinical effects of NF (which also include unspecific effects) may have been underestimated. NF-studies in ADHD with waiting list controls tend to yield much higher effect sizes than those with active or semi-active control conditions (see Cortese et al., 2016). In addition, none of the NF studies published so far used the rather conservative BOCF method. Therefore, a comparison with those studies should consider the medium ES for the completers. Furthermore, it must be noted that a meta-analysis of cognitive trainings in ADHD yielded an ES of 0.37 (Cortese et al., 2015). Similarly, for behavioral interventions the meta-analysis reported an ES of 0.35 (Daley et al., 2014). In addition, we analyzed *post hoc* within ES for our groups. Here, medium to large effect sizes of 0.78 (BOCF) and 1.09 (completer) for NF were observed, while the effect sizes for EMG were small with 0.35 (BOCF) and 0.48 (completer).

According to teachers, who can be regarded as possibly blinded raters, there was no group effect in favor of NF. This is of considerable concern in the light of a recent meta-analysis highlighting smaller effect sizes when applying probably blinded vs. non-blinded ratings (Cortese et al., 2016). Whether NF helps more or faster in the home setting than in school or whether teachers are less sensitive to change than parents are still unresolved questions.

Similar to findings from other ADHD studies (e.g., Sollie et al., 2013), teachers compared to parents rated children as being less affected. This may have contributed to the non-significant findings since more pronounced baseline ADHD symptoms were associated with a better response to NF. Within-group analysis of our teachers’ results revealed effects of NF on the global ADHD score, inattention, and impulsivity, while EMG feedback did not result in such significant changes. A recent meta-analysis showed similar results of NF based on teacher ratings on inattention (Micoulaud-Franchi et al., 2014). Unfortunately, our study was not powered to detect differences between treatments based on teacher ratings, but the small effect sizes could also suggest that the SCP-NF specific improvements may be of limited significance in school settings. This raises the possibility that more training sessions and transfer trials, or more sensitive blinded ratings may be needed for SCP-NF to produce clinically significant improvement of ADHD symptoms in school settings. However, the observation that teachers judged the children as significantly less affected may put these considerations into a different perspective. If there is less clinical relevance perceived there may be less need for and awareness of change. As discussed by Cortese et al. (2016), teachers may be less attentive to improvements or the instruments used should be complemented, e.g., by behavior observation. Furthermore, teachers’ ratings being probably blind regarding treatment allocation are not necessarily more precise. Blinding does not validate ratings as superior *per se*. Recently, Janssen et al. (2016) reported reductions in theta power that were predictive of parents’ ratings of reduced inattention, whereas no such association was found for (probable blinded) teachers’ ratings.

Physicians or clinical psychologists not involved in the study rated about 27% of children in both groups as responders based on CGI ratings. The almost identical response rate in both groups supports the assumption of large unspecific effects of the treatments. Unfortunately, there were about 40% missing values. Furthermore, the validity of the clinicians’ ratings is questionable, as some parents reported that the clinicians asked them about their own judgment and gave their ratings accordingly. To supplement the response ratings of clinicians, we determined how many parents described a reduction of ADHD total symptoms of more than 25% for their child. Here, 52% of NF and 35% of EMG children (mITT population) were rated as improved. This result is comparable to response rates reported by Gevensleben et al. (2009) with 52% for NF and 29% for the computer based attention skills training.

The *a priori* decision to define parents’ ratings as “primary” was not only based on methodological requirements. Parents observe many facets of their children’s everyday family, social and academic life, and suffer from impairment in all these areas. This may not only explain the more severe ratings compared to those given by teachers but also points to the ecological validity of their judgments. Although parents were not informed about treatment allocation, we cannot rule out that information given to them by their children may have affected their ratings. Parents’ ratings were probably not blinded because children were instructed differently according to treatment allocation. Blinding of patients and staff may count as a gold standard of evidence-based medicine in drug research but may interfere in treatments where patients are expected to learn a certain behavior or skill. This holds true for psychotherapy in general and it is of utmost importance in feedback therapy aiming at the acquisition of a self-regulation skill. Without knowing which parameter has to be trained the patient may lose time, motivation and precision (Surwit and Keefe, 1983). An important feature even in blinded designs is the control of expectations influencing the outcome of any treatment (Benedetti et al., 2005; Oken, 2008). In our study, parents’ expectations had no effect on outcome. However, their satisfaction was high and did not differ between treatments, again pointing to the impact of unspecific variables acting similarly in both groups. The assessment of expectations is a first step although the psychometric quality of the questionnaire we used is not yet assessed. We also considered that alternative control conditions where EEG activity unrelated to ADHD must be regulated could have reduced perceptual awareness and allowed blinding. However, we were not aware of any EEG activity that is completely unrelated to ADHD on the one hand and would do no potential harm on the other hand.

In addition to comparing the reduction of symptoms between groups self-regulation performance and its correlation with clinical outcome was analyzed. This analysis yielded mixed results: in the absence of significant correlations between self-regulation and clinical outcome (global score) the (amount of) specificity remains questionable. On the other hand, more children in the EMG group than in the NF-group learned to improve self-regulation, consistent with the results of Maurizio et al. (2014). Subsequently, better self-regulation and learning resulted in more positive reinforcement (i.e., more frequent reinforcement following successful trials) for children of the EMG group. As learning to self-regulate is acknowledged as an important unspecific variable in biofeedback, one could have expected more clinical improvement and superior outcomes in the EMG group. This was not the case. Therefore, the clinical advantage of NF is unlikely due to unspecific effects only. Given the many ways of analyzing learning (e.g., within sessions learning vs cross sessions as well as pre–post differences in spontaneous as well as event-related brain activity; Gruzelier, 2014; Maurizio et al., 2014; Zuberer et al., 2015) further analyses, including follow-up observations will give more insight in this important matter.

For the first time, AE and SAE of SCP-NF were investigated with the help of the WHO Adverse Reaction Terminology, included in the Medical Dictionary for Regulatory Activities for clinical studies (MedDRA^®^, Version 16). The treatments were feasible and AE related to the treatment were observed in only a few children. While one child of the EMG group had to be withdrawn from the study because his symptoms deteriorated, the other AE in children of both groups remitted quickly.

The drop-out rate was similar to previous NF-studies with comparable duration of treatment. Most drop-outs were observed between pre-test and end of first treatment phase. In accordance with evidence on ADHD treatment utilization adherence may have been hampered by personal and family characteristics of dropouts (higher level of oppositional symptoms, lower paternal level of education, more single parents) (Corkum et al., 2015). Such families may require special attention when behavioral interventions are planned.

A difference in the change of the full scale IQ was observed between groups. While there was a slight increase in NF, performance of EMG participants declined. This may be due to EMG children investing less effort in the test, and to SCP-NF releasing attentional resources (Strehl et al., 2011).

Earlier studies have already reported improvements in children with ADHD after SCP-NF. We have moved a step forward in answering questions regarding specificity, efficacy and feasibility with this study. We included the largest sample treated with NF to date, used a semi-active control condition with an identical setting, a conservative statistical approach (BOCF), and SCPs as target for NF, which has been identified as a stable marker of ADHD. Major limitations of the present study are the lack of power regarding teacher ratings, and only few and questionable clinicians’ ratings. Compared with other studies, a possible shortcoming might lie in the fact that for pragmatic reasons, we chose to conduct only 25 training sessions since Arns et al. (2009) observed a correlation of the number of sessions with the reduction of inattention. More sessions and more transfer trials might have improved performance in those trials and clinical effects might have become more robust.

Further analysis of electrophysiological and neuropsychological data and long-term outcome will help to understand the mechanisms underlying the reported specific and unspecific effects. A major challenge for future studies will be to identify predictors to decide whether an individual patient would particularly benefit from SCP-NF.

## Author Contributions

MH, US, and DW conceived and designed the trial. MH and US coordinated the trial. MH, US, SW, and MA designed and supervised the intervention component. MA, CB, LH, TB, SG, LH, SH, AM-M, SM, BP, SW, and YF conducted training sessions and pre-/post-measurements. DW and CR conducted the analyses of behavioral data, PA and DB the analysis of EEG data. US and MH interpreted the results and drafted the report. All authors revised the article for important intellectual content. MH and US are the principal investigators.

## Conflict of Interest Statement

AR is member of an advisory board and speakers’ bureau of Lilly, Shire, Medice, and Novartis. He has received research and travel support and an educational grant from Shire. CF has received speaker’s fees from Eli Lilly and Shire. DW has been employed at Boehringer Ingelheim since January 2015. His study specific contribution as the responsible statistician took place when he was employed at IZKS Mainz. TB has served in an advisory or consultancy role for Hexal Pharma, Lilly, Medice, Novartis, Otsuka, Oxford Outcomes, PCM Scientific, Shire, and Vifor Pharma. He has received conference attendance support and conference support or received speaker’s fees from Lilly, Medice, Novartis, and Shire. He is/has been involved in clinical trials conducted by Lilly, Shire, and Vifor Pharma. MH has served in an advisory or consultancy role for Medice and Shire, and has received conference attendance support or was paid for public speaking by Lilly, Medice, neuroConn, and Shire. US has been paid for public speaking by Novartis, Medice, neuroCare, the German Society for Biofeedback, and Akademie König und Müller. DB serves as an unpaid scientific consultant of an EU-funded neurofeedback trial. The other authors declare that the research was conducted in the absence of any commercial or financial relationships that could be construed as a potential conflict of interest.

## Abbreviations

AE: adverse events
CBCL: Child Behavior Checklist
CGI-I: Clinical Global Impression-Improvement Scale
CPM: Coloured Progressive Matrices
EMG: electromyographic feedback
EOG: electrooculogram
IZKS: Interdisciplinary Center for Clinical Trials
mITT: modified intention-to-treat (population)
NF: neurofeedback
PP: per-protocol (population)
SAE: serious adverse events
SCP: slow cortical potential
SDQ: Strengths and Difficulties Questionnaire.
