# Supplementary material for: Neurofeedback of Slow Cortical Potentials in Children with Attention-Deficit/Hyperactivity Disorder: A Multicenter Randomized Trial Controlling for Unspecific Effects
Source: Front Hum Neurosci. 2017 Mar 31;11:135. doi: 10.3389/fnhum.2017.00135 (PMC5374218; doi:10.3389/fnhum.2017.00135)
Supplement: Supplementary file 1 [file Table_1.DOCX]

**Appendix Table S1: Sensitivity Analysis: Differences in FBB-ADHS global score (Parents’ ratings; Post-Test 2 minus Pretest between groups; PP Population; ANCOVA, BOCF)**

|  | **Adjusted mean (95% CI)** | **p-value** |
| --- | --- | --- |
| EMG-Feedback | -0.1074 ( (-0.2774 / 0.0626) |  |
| Neurofeedback | -0.3287 (-0.4806 / -0.1769) |  |
| Difference between treatments | 0.2213 (0.0293 / 0.4133) |  |
| Treatment |  | 0.0245 |
| Baseline FBB-ADHS global score |  | 0.0042 |
| Gender |  | 0.1199 |
| Trial site |  | 0.9972 |
| Baseline ADHD medication (yes/no) |  | 0.7604 |
| Parenting style |  | 0.6001 |
| Parents’ expectations |  | 0.9069 |
