# Supplementary material for: Neurofeedback of Slow Cortical Potentials in Children with Attention-Deficit/Hyperactivity Disorder: A Multicenter Randomized Trial Controlling for Unspecific Effects
Source: Front Hum Neurosci. 2017 Mar 31;11:135. doi: 10.3389/fnhum.2017.00135 (PMC5374218; doi:10.3389/fnhum.2017.00135)
Supplement: Supplementary file 2 [file Table_2.DOCX]

**Appendix Table S2 Parents’ ADHD Ratings (mITT Population N=144)**

|  | **NF** | | **EMG** | | **Total** | |
| --- | --- | --- | --- | --- | --- | --- |
|  | Pre-Test | Post-Test 2 | Pre-Test | Post-Test 2 | Pre-Test | Post-Test 2 |
| **Hyperactivity** | | | | | | |
| N | 72 | 53 | 67 | 50 | 139 | 103 |
| Mean (SD) | 1.543 (0.628) | 1.086 (0·689) | 1.524 (0.665) | 1.265 (0.664) | 1.534 (0·644) | 1.173 (0·679) |
| Missing | 3 | 22 | 2 | 19 | 5 | 41 |
| **Impulsivity** | | | | | | |
| N | 72 | 53 | 67 | 50 | 139 | 103 |
| Mean (SD) | 1.927 (0.690) | 1.453 (0.574) | 1.799 (0.779) | 1.685 (0.779) | 1.865 (0.734) | 1.566 (0.688) |
| Missing | 3 | 22 | 2 | 19 | 5 | 41 |
| **Inattention** | | | | | | |
| N | 72 | 53 | 67 | 50 | 139 | 103 |
| Mean (SD) | 2.033 (0.527) | 1.499 (0·534) | 1.973 (0.509) | 1.705 (0.448) | 2.004 (0.518) | 1.599 (0.502) |
| Missing | 3 | 22 | 5 | 19 | 5 | 41 |
| **Global Score*** | | | | | | |
| N | 73 | 53 | 67 | 50 | 139 | 103 |
| Mean (SD) | 1.842 (0.448) | 1.346 (0.519) | 1.782 (0.471) | 1.548 (0.488) | 1.813 (0.459) | 1.444 (0.512) |
| Missing | 3 | 22 | 2 | 19 | 5 | 41 |

*Global score could not be assessed if more than 2 items of subscales were missing
