# Supplementary material for: Neurofeedback of Slow Cortical Potentials in Children with Attention-Deficit/Hyperactivity Disorder: A Multicenter Randomized Trial Controlling for Unspecific Effects
Source: Front Hum Neurosci. 2017 Mar 31;11:135. doi: 10.3389/fnhum.2017.00135 (PMC5374218; doi:10.3389/fnhum.2017.00135)
Supplement: Supplementary file 3 [file Table_3.DOCX]

**Appendix Table S3: Differences in FBB-ADHS global score (Teachers’ ratings; Post-Test 2 minus Pretest between groups; mITT population, ANCOVA, BOCF)**

|  | **Adjusted mean (95% CI)** | **p-value** |
| --- | --- | --- |
| EMG-Feedback | -0.1134 (-0.2628 / 0.0360) |  |
| Neurofeedback | -0.1549 (-0.2953 / -0.0145) |  |
| Difference between treatments | 0.0415 (-0.1240 /0.2070) |  |
| Treatment |  | 0.6204 |
| Baseline FBB-ADHS global score |  | **<.0001** |
| Gender |  | 0.9686 |
| Trial site |  | 0.2200 |
| Baseline ADHD medication (yes/no) |  | 0.8498 |
| Parenting style |  | 0.6290 |
| Parents’ expectations |  | 0.5949 |
