# Supplementary material for: Neurofeedback of Slow Cortical Potentials in Children with Attention-Deficit/Hyperactivity Disorder: A Multicenter Randomized Trial Controlling for Unspecific Effects
Source: Front Hum Neurosci. 2017 Mar 31;11:135. doi: 10.3389/fnhum.2017.00135 (PMC5374218; doi:10.3389/fnhum.2017.00135)
Supplement: Supplementary file 4 [file Table_4.DOCX]

**Appendix Table S4 Teachers’ ADHD Ratings (mITT Population N=144)**

|  | **NF** | | **EMG** | | **Total** | |
| --- | --- | --- | --- | --- | --- | --- |
|  | **Pre-Test** | **Post-Test 2** | **Pre-Test** | **Post-Test 2** | **Pre-Test** | **Post-Test 2** |
| **Hyperactivity** | | | | | | |
| N | 63 | 51 | 68 | 42 | 131 | 93 |
| Mean (SD) | 1.147 (0.812) | 1.073 (0.810) | 1.024 (0.854) | 0.954 (0.735) | 1.088 (0.831) | 1.019 (0.775) |
| Missing | 7 | 24 | 6 | 27 | 13 | 51 |
| **Impulsivity** | | | | | | |
| N | 68 | 51 | 63 | 42 | 131 | 93 |
| Mean (SD) | 1.412 (0.954) | 1.270 (0.963) | 1.310 (0.954) | 1.298 (0.926) | 1.363 (0.952) | 1.282 (0.941) |
| Missing | 7 | 24 | 6 | 27 | 13 | 51 |
| **Inattention** | | | | | | |
| N | 68 | 51 | 63 | 42 | 131 | 93 |
| Mean (SD) | 1.693 (0·696) | 1.595 (0.765) | 1.676 (0.724) | 1.468 (0.627) | 1.685 (0.707) | 1.538 (0.705) |
| Missing | 7 | 24 | 6 | 27 | 13 | 51 |
| **Global Score*** | | | | | | |
| N | 65 | 51 | 60 | 40 | 125 | 91 |
| Mean (SD) | 1.479 (0.637) | 1.348 (0.732) | 1.381 (0.709) | 1.242 (0.634) | 1.432 (0.671) | 1.302 (0.689) |
| Missing | 10 | 24 | 9 | 29 | 19 | 53 |

* Global score could not be assessed if more than 2 items of subscales were missing
